# Type VI Secretion Systems Present New Insights on Pathogenic *Yersinia*

**DOI:** 10.3389/fcimb.2018.00260

**Published:** 2018-07-31

**Authors:** Xiaobing Yang, Junfeng Pan, Yao Wang, Xihui Shen

**Affiliations:** ^1^State Key Laboratory of Crop Stress Biology for Arid Areas, College of Life Sciences, Northwest A&F University, Yangling, China; ^2^Shaanxi Key Laboratory of Agricultural and Environmental Microbiology, College of Life Sciences, Northwest A&F University, Yangling, China

**Keywords:** pathogenic *Yersinia*, type VI secretion system (T6SS), function, effectors, regulation

## Abstract

The type VI secretion system (T6SS) is a versatile secretion system widely distributed in Gram-negative bacteria that delivers multiple effector proteins into either prokaryotic or eukaryotic cells, or into the extracellular milieu. T6SS participates in various physiological processes including bacterial competition, host infection, and stress response. Three pathogenic *Yersinia* species, namely *Yersinia pestis, Yersinia pseudotuberculosis*, and *Yersinia enterocolitica*, possess different copies of T6SSs with distinct biological functions. This review summarizes the pathogenic, antibacterial, and stress-resistant roles of T6SS in *Yersinia* and the ion-transporting ability in *Y. pseudotuberculosis*. In addition, the T6SS-related effectors and regulators identified in *Yersinia* are discussed.

## Introduction

The genus *Yersinia* comprises three species of bacterial pathogens, namely *Y. pestis, Y. enterocolitica*, and *Y. pseudotuberculosis*, that are causative agents of human diseases. *Y. pestis* is the etiological agent of plague, often transmitted by flea bites or aerosols. It infects regional lymph nodes or lungs and causes the highly lethal disease in humans. *Y. pseudotuberculosis* and *Y. enterocolitica* are enteric pathogens, which usually grow in the environment and can be transmitted to mammalian hosts through ingestion of contaminated food or water. They typically cause a broad range of gastrointestinal diseases, from enteritis to mesenteric lymphadenitis (Bibikova, 1977; Brubaker, 1991; Bottone, 1997; Putzker et al., 2001; Pujol and Bliska, 2005). These three bacterial species have been used in experimental models of infection to study their pathogenicity for the mammalian host.

Despite differences in pathogenesis, these virulent *Yersinia* species have several common virulence factors, including the 70-kb virulence plasmid (pCD1 in *Y. pestis* and pYV in enteropathogenic *Yersinia*) and the yersiniabactin (Ybt) system (Brubaker, 1991; Heesemann et al., 1993; Cornelis et al., 1998). The 70-kb plasmid contains dozens of genes encoding structural components of a type III secretion system (T3SS), and also encodes several T3SS effector proteins called *Yersinia* outer proteins (Yops) and their dedicated chaperones to subvert the innate immune system of the hosts (Bliska et al., 2013; Schwiesow et al., 2015). Since enzymes/toxins/effectors delivered by secretion systems play a crucial role in the interaction between pathogens and their hosts or competitors, the various types of secretion systems attract interest in the research on pathogenic bacteria.

Several virulence associated secretion (*vas*) genes in T6SS gene clusters were identified in bacterial pathogens before, but till the year of 2006 the Type VI secretion system was proposed and defined as a contact-dependent secretion mechanism (Mougous et al., 2006; Pukatzki et al., 2006). T6SS was found distributed in about 25% of all sequenced Gram-negative bacteria with high conservation (Boyer et al., 2009; Salomon and Orth, 2015). The T6SS is a proteinaceous machinery that directly injects effector proteins into target cells in a one-step process with a bacteriophage-like cell-puncturing device (Zoued et al., 2014), although the membrane puncturing process has not been directly observed to date. The versatile T6SS weapons allow bacteria to compete with other bacteria or attack simple or higher eukaryotes. The main function of T6SS was implicated in virulence, commensalism or symbiosis, and interbacterial competition (Jani and Cotter, 2010). Apart from the conserved components and similar structures, various functions of T6SSs have been exploited in the past decade, and the newly discovered effectors and their dedicated chaperones secreted by T6SS are also being updated. In the three pathogenic *Yersinia* species, different series of T6SS seem to possess different functions. This review focuses on the detailed description of T6SSs function in *Yersinia*.

## Comparison of T6SS gene clusters in *yersinia* species

### Component, structure, and energetics of T6SSs

The core components of T6SS contain 13 subunits, which comprise the typical T6SS structure similar to the T4 bacteriophage, with tail, spike, sheath, hub or baseplate proteins (Boyer et al., 2009; Cascales and Cambillau, 2012). The typical T6SS structure is composed of three subunits: the membrane complex, the baseplate complex, and the injection apparatus. The membrane complex is composed of proteins TssJLM, which anchor the baseplate complex (TssAEFGK) to the membrane and provide structural support. The injection apparatus contains needle sheath (TssBC), tail tube (TssD/Hcp) and spike complex (TssI/VgrG and PAAR motifs) (Silverman et al., 2012; Brunet et al., 2015; Cianfanelli et al., 2016). Generally, various effectors and chaperones could bind to this injection apparatus when they were needed to be secreted out through the T6SS apparatus, and the secretion of Hcp or VgrG is often regarded as the hallmark of a functional T6SS in many bacterial species (Mougous et al., 2006; Pukatzki et al., 2006; Wang et al., 2011). The PAAR (Proline-Alanine-Alanine-aRginine) repeat containing proteins were regarded as an additional component of the T6SS machinery. PAAR-repeat could form a sharp conical extension on the tip of the VgrG spike, which is further involved in the effector recruitment and attaching effector domains to the spike (Shneider et al., 2013; Bondage et al., 2016). The 13 core components of typical T6SS are listed in Table 1.

**Table 1:** The 13 conserved components of typical T6SS.

**COG No**.	**Protein name**	**Alternative names**	**Location/activity**	**Homologous proteins**
COG3515	TssA	BimE	Baseplate	
COG3516	TssB	VipA/iglA	Contractile sheath	phage T4 gp18
COG3517	TssC	VipB/iglB	Contractile sheath	phage T4 gp18
COG3157	TssD	Hcp	Form the secreted hexameric tube	phage T4 gp19
COG3518	TssE	HsiF	Baseplate	phage T4 gp25
COG3519	TssF	VasA	Baseplate	
COG3520	TssG		Baseplate	
COG0542	TssH	ClpV	Sheath recycling AAA+ ATPase	
COG3501	TssI	VgrG	Tail spike	phage T4 gp27/5
COG3521	TssJ	Lip, SciN	Membrane anchoring complex	
COG3522	TssK		Baseplate	
COG3455	TssL	IcmH/DotU, VasF	Membrane anchoring complex	T4bSS IcmH
COG3523	TssM	IcmF, VasK	Membrane anchoring complex	T4bSS IcmF

The secretion of T6SS is a dynamic cycle of assembly/extension, contraction/puncture, and disassembly of the sheath, accompanying the energy supply and effector transportation (Basler et al., 2012). The initial stage of the T6SS assembly is to form the membrane complex, followed by the recruitment of baseplate proteins for anchor and extension of the outer sheath and the inner tube (Brunet et al., 2015). This indicated that the T6SS shares a common assembly pathway of tail tubes with bacteriophage (Brunet et al., 2014). During the attack process, the sheath-like structure propels an inner tube through contraction, and the membrane-puncturing spike is pierced into the target cells. Then effectors and chaperones are delivered into target cells with this expelled structure in a one-step manner (Cianfanelli et al., 2016).

### Different series of T6SS in three *yersinia* species

**Figure 1:**
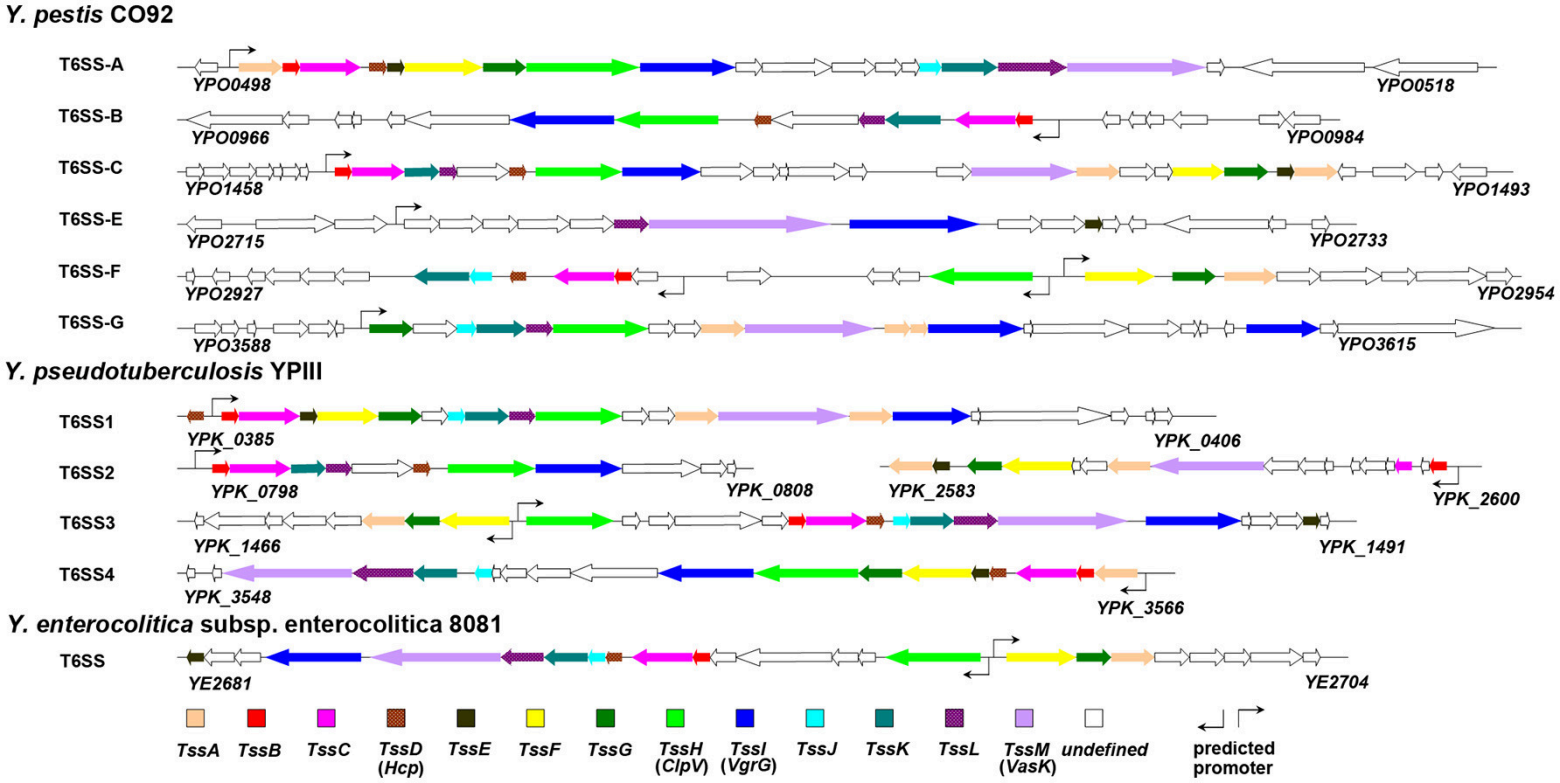
T6SS gene clusters in three pathogenic *Yersinia* species.

T6SS displays a single copy in majority of the bacterial species, yet multiple distinct copies are found in several bacterial species (Boyer et al., 2009). In the *Yersinia* species, six and four T6SS clusters were identified in *Y. pestis* (Andersson et al., 2017) and *Y. pseudotuberculosis* (Zhang et al., 2011b), respectively, while only one copy was found in *Y. enterocolitica* (Jaakkola et al., 2015). The T6SS gene clusters in *Yersinia* are shown in Figure 1.

**Figure 2:**
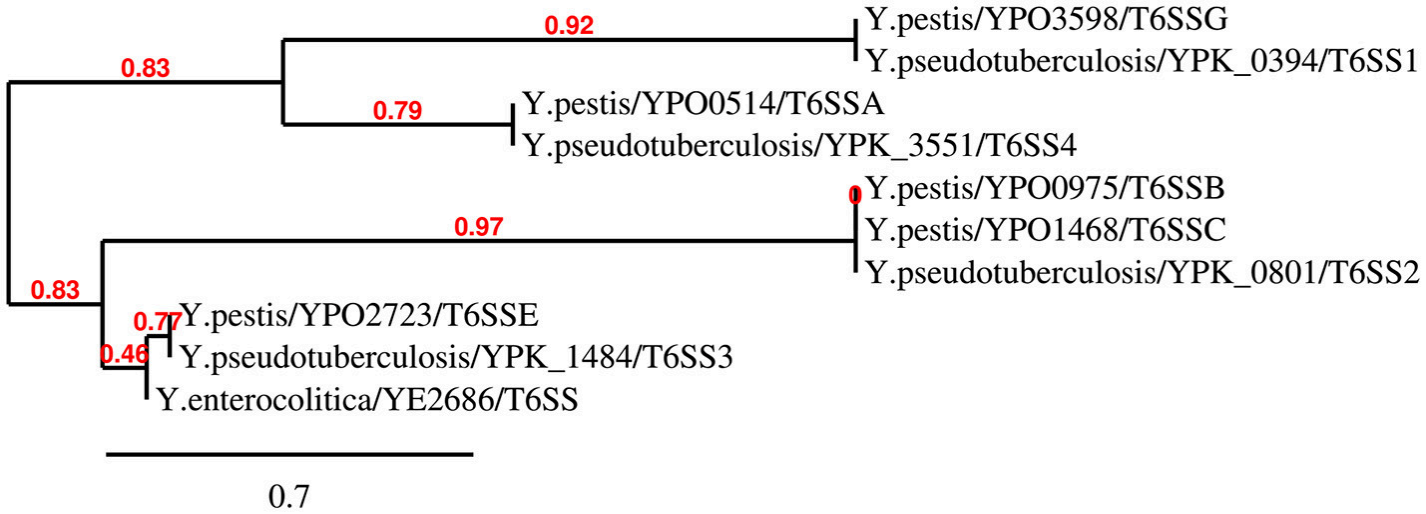
Neighbour-Joining tree of TssL proteins of *Yersinia* T6SSs. The phylogenetic tree was generated using BioNJ with 100 bootstrap replicates based on the alignment of TssL sequences (Gascuel, 1997). As the *TssL* is missed in T6SS gene cluster F in *Y. pestis*, the T6SS gene cluster F was not present in this N-J dendrogram.

It is noteworthy that the multiple distinct T6SS copies are not functionally redundant; for example, T6SS-1 and T6SS-5 in *B. thailandensis* mediate the bacterial antagonism and macrophage infection, respectively (Schwarz et al., 2010). In *Y. pestis*, the T6SS region *YPO0498-YPO0516* (the T6SS Cluster A in Figure 1) is preferentially expressed at 26 vs. 37°C (Cathelyn et al., 2006; Robinson et al., 2009), which suggests this gene cluster may function in natural conditions rather than in its mammalian host. Deletion of the T6SS Cluster A locus reduced the uptake by J774.1 murine macrophages (Robinson et al., 2009). However, it had no effect on virulence in bubonic or pneumonic murine plague models compared to the parental *Y. pestis* CO92 strain (Robinson et al., 2009; Andersson et al., 2017). An Hcp-like protein encoded by *ypo0502* was found to play important roles in autoagglutination (AA), indicating that the T6SS Cluster A is involved in intraspecies interaction of bacteria (Podladchikova et al., 2011). A similar thermoregulated gene cluster in *Y. pseudotuberculosis* was identified as T6SS4 (*ypk_3548*- *ypk_3566*), which is precisely regulated by temperature, growth phase, and AHL-dependent quorum sensing systems, and plays a crucial role in resistance to environmental stresses (Zhang et al., 2011b). The T6SS Cluster A in *Y. pestis* and T6SS4 in *Y. pseudotuberculosis* could both be induced by room temperature conditions (about 25°C). The phylogenetic analysis based on TssL of *Yersinia* T6SSs showed a close genetic distance between T6SS Cluster A in *Y. pestis* and T6SS4 in *Y. pseudotuberculosis* (Figure 2). Furthermore, the two T6SS clusters have similar genomic organization, suggesting they may both play crucial roles in environment adaptability. From Figure 1 it showed the T6SS-E and F clusters in *Y. pestis* only contain 4 and 9 T6SS genes, respectively. Considering these components seemed complementary, and the two T6SS clusters locate in vicinity to each other, it speculated they may represent an integral set of T6SS. It is worth noting that *Y. pestis* has 6 clusters but only one with a complete set of T6SS genes, while *Y. pseudotuberculosis* has 4 clusters with full set of genes. Note that some clusters encode an incomplete set of T6SS genes and could be non-functional. The function of these incomplete T6SSs should be experimental verified in the future.

## The functions of T6SS in *yersinia*

### Virulence

At the beginning, genes of T6SS cluster in *Vibrio cholerae* and *Pseudomonas aeruginosa* were identified as *vas* (*v*irulence-*a*ssociated *s*ecretion) genes for their roles in pathogenesis (Mougous et al., 2006; Pukatzki et al., 2006). Afterwards, the virulence-related functions of T6SS in the host were verified in several organisms including *Edwardsiella tarda* (Zheng and Leung, 2007), *Aeromonas hydrophila* (Suarez et al., 2008), *Salmonella enterica* (Blondel et al., 2010), *Campylobacter jejuni* (Lertpiriyapong et al., 2012), and *Burkholderia pseudomallei* (Schwarz et al., 2014).

In 2008, five T6SS gene clusters in *Y. pestis* KIM were identified through a phylogenetic analysis (Bingle et al., 2008), while six T6SS clusters in *Y. pestis* CO92 were predicted based on *in silico* analysis (Boyer et al., 2009). Actually, six T6SS clusters are distributed in genome of *Y. pestis* KIM and CO92 according to the SecReT6 database, which is a web-based resource for type VI secretion systems found in bacteria (Li et al., 2015). However, the virulence of T6SS in *Y. pestis* to hosts was confirmed through laboratory experiments till 2015. Using an *in vivo* signature-tagged mutagenesis (STM) screening approach, Ponnusamy et al. identified three T6SS genes with virulence potential in *Y. pestis* CO92. In-frame deletion of *vasK* (*YPO3603*, a component of the T6SS cluster G) in *Y. pestis* CO92 resulted in significant attenuation of the bacterium in murine models of infection, and this attenuation could be fully complemented (Ponnusamy et al., 2015). In addition, the five deletion mutants of T6SS clusters (B, C, E-G) showed varying levels of attenuation when evaluated in a mouse model of pneumonic plague, but not all T6SS clusters of *Y. pestis* are equally functional. Deletion mutants for Cluster C and Cluster F exhibited limited, 14%, or no attenuation, respectively, while deletion mutants for Cluster B, Cluster E, and Cluster G, exhibited significant levels of attenuation in infection of a bubonic plague model. A further augmented attenuation was detected in mutants lacking multiple T6SSs. The Δ*ypo2720-2733*Δ*hcp3* (Cluster E and *hcp* homolog 3) double deletion mutant showed 60% of attenuation in comparison to WT CO92. This result further supports the involvement of T6SSs in *Y. pestis* virulence (Andersson et al., 2017). Moreover, most of the attenuated T6SS deletion mutant strains showed decreased host cell cytotoxicity and intracellular survival in comparison to WT CO92 (Andersson et al., 2017).

*Y. pseudotuberculosis* YPIII mutants lacking T6SS-4 or *yezP* (*ypk_3549*, an effector gene in T6SS-4 cluster) are defective in virulence in mice (Wang et al., 2015). The enteropathogenic *Y. enterocolitica* causes infections similar to *Y. pseudotuberculosis*, but has several different characteristics related to epidemiology and ecology, and only one type of T6SS was found in the *Y. enterocolitica* genome. It is speculated that the lack of multiple T6SS might be beneficial for escaping the host immune system, because a T6SS with toxic effectors might encumber the invasion and survival of *Y. enterocolitica* cells in mammalian hosts (Jaakkola et al., 2015). This function can be regarded as anti-virulence, which has been discussed previously (Jani and Cotter, 2010).

### Bacteria-bacteria interaction

Bacteria have evolved various mechanisms to kill bacterial competitors and defense against predators in their environment. The T6SS has membrane-penetrating tails similar to bacteriophages, suggesting that T6SS may be involved in cell-cell interactions of bacteria (Pell et al., 2009). Bacteria could utilize T6SS to intimately interface with other bacteria, efficiently killing or inhibiting competitors with T6SS toxins and protecting itself with immune proteins, which has been reported in *P. aeruginosa* (HSI-1) (Hood et al., 2010), *B. thailandensis* (T6SS-1) (Schwarz et al., 2010), *V. cholerae* (MacIntyre et al., 2010; Ishikawa et al., 2012), and *Serratia marcescens* (Murdoch et al., 2011). Different from the traditional offensive and defensive model of T6SS mediated bacterial competition (Russell et al., 2012; Lien and Lai, 2017; Yang et al., 2018), swarming *Proteus mirabilis* discriminate non-identical population via T6SS-dependent delivery of toxic effectors, thus forming a visible demarcation line (Dienes line) between different *Proteus* isolates (Alteri et al., 2013). Although this phenomenon of self-recognition during swarming in *P. mirabilis* has been known for decades it was only ascribed to the T6SS recently.

Through DNA microarrays analysis of *Y. pestis* CO92, an IAHP (*I*cmF-*A*ssociated *H*omologous *P*rotein) locus (*YPO0498-0516*) was found regulated by RovA, which was specifically required for bubonic plague (Cathelyn et al., 2006). However, another study indicated that mutation in this locus had no effect in laboratory models of infected oriental rat flea *X. cheopis*, or in virulence using bubonic or pneumonic murine plague models (Robinson et al., 2009). Similarly, in-frame deletion of *YPO0498* did not affect virulence *in vivo* (Ponnusamy et al., 2015). Recently, this locus was classified as T6SS cluster A in *Y. pestis* CO92, and was confirmed not required for pathogenesis (Andersson et al., 2017). Nevertheless, deletion of the T6SS Cluster A locus resulted in increased uptake and intracellular growth of *Y. pestis* in the macrophage-like J774.A1 cells, indicating a role of T6SS in interaction with host cells (Robinson et al., 2009). Among this T6SS cluster, YPO0502, identified as a Hcp-like protein that forms pilus-like structure, determines *Y. pestis* autoagglutination (AA). This suggests that at least one (T6SS Cluster A) of the 6 T6SS clusters in *Y. pestis* is involved in bacterial interaction (Podladchikova et al., 2011). However, whether Yersinia T6SSs are involved in bacterial competition and Dienes line formation needs to be verified in the future.

### Stress response

Many of the earliest studies on T6SS focused on their roles in virulence and the interactions between pathogens and hosts. In 2009, it was reported that a T6SS in *Vibrio anguillarum* acts as a sensor for an unknown extracytoplasmic signal that modulates RpoS (Weber et al., 2009). RpoS is the main regulator of gene expression during stationary phase and stress conditions, and it positively regulates VanT linking quorum sensing (QS) to stress response and general physiology of *V. anguillarum* (Weber et al., 2008). Therefore, this study suggested a new function for T6SS in the ecology of bacteria, that T6SS played a crucial role in bacterial stress response and cell survival after exposure to various environmental challenges (Weber et al., 2009).

In *Y. pseudotuberculosis*, RpoS was found to be important in resistance to multiple stressors including oxidative stress, acid stress, osmotic stress, and 42°C heat shock, and was also crucial for motility, biofilm formation, and T6SS expression. The electrophoretic mobility shift assay (EMSA) showed that RpoS regulates T6SS4 expression by directly binding to the T6SS4 promoter (Guan et al., 2015). However, compared with the two reported YpsRI QS system (Atkinson et al., 2008) and the OmpR regulator (Zhang et al., 2013), RpoS plays a less crucial role in T6SS4 activation. T6SS4 is only partly responsible for the stress-resistance activity of RpoS, consistent with the relatively weak role of RpoS in activating T6SS4 expression in *Y. pseudotuberculosis* (Guan et al., 2015). Similarly, the PppA-PpkA pair of H1-T6SS in *P. aeruginosa* plays roles in bacterial resistance to oxidative and osmotic stress, and *pppA-ppkA* deletion affects the expression of the RpoS and QS regulons (Goldova et al., 2011).

In 2013, an OmpR-T6SS4 regulation pathway for stress resistance in *Y. pseudotuberculosis* was characterized in two studies, both of which confirmed the direct binding of OmpR on the T6SS-4 promoter region *in vitro* (Gueguen et al., 2013; Zhang et al., 2013). In an earlier study, OmpR was found to be essential for low pH adaptation by positively regulating the expression of urease in *Y. pseudotuberculosis* (Hu et al., 2009). Here, it was indicated that T6SS4 contributed to acid survival by maintaining a steady-state intracellular pH in *Y. pseudotuberculosis*. The acid resistance ability is dependent upon the ATPase activity of ClpV4 that participates in H^+^ extrusion, and this process is positively regulated by the global regulator OmpR (Zhang et al., 2013).

In addition, the T6SS-4 expression mediated by OmpR is also induced in high osmolarity conditions or in the presence of sodium deoxycholate. Besides T6SS4 gene cluster, OmpR activates additional genes involved in tolerance to high osmolarity, because the *ompR* transposon mutant cells were more severely affected than the *tssF4* cells (Gueguen et al., 2013). Similarly, an earlier study showed that deletion of T6SS increased the susceptibility of *C. jejuni* to a bile salt, deoxycholic acid (DCA), and the reason for which was attributed to an increase in the intracellular influx of DCA mediated by T6SS (Lertpiriyapong et al., 2012).

### Ion transport

Recently, T6SS4 in *Y. pseudotuberculosis* was found to have a notable function of transporting zinc ions (Zn^2+^) from the environment into bacterial cells to mitigate the detrimental hydroxyl radicals induced by multiple stressors (Wang et al., 2015). Generally, bacteria utilize the classical ZnuABC transport system to acquire Zn^2+^ to maintain zinc balance. In *Y. pseudotuberculosis*, the ZnuABC transporter contributes to reduce the intracellular ROS levels and thus prevents oxidative damage to cells (Wang et al., 2016). While in *Y. pestis*, the yersiniabactin (Ybt, a zincophore for Zn^2+^ acquisition) synthetase HMWP2, together with ZnuABC, are critical for lethal infections in a mouse model of septicemic plague (Bobrov et al., 2014). Interestingly, in *Y. pseudotuberculosis* a T6SS4-mediated substrate YezP (YPK_3549), a novel Zn^2+^-binding protein, has the capacity to rescue the sensitivity to oxidative stress exhibited by T6SS4 mutants when added to extracellular milieu. Zn^2+^ acquisition achieved by YezP is co-regulated with T6SS4 by OxyR, which is a global oxidative stress regulator that senses diverse environmental cues (Wang et al., 2015).

Similar capability of ion transport by T6SS was found in *B. thailandensis* and *P. aeruginosa*. In *B. thailandensis*, a proteinaceous zincophore (TseZ) secreted through T6SS4 interacts with the outer membrane heme transporter HmuR to acquire zinc under oxidative stress (Si et al., 2017a). In addition, the T6SS4 in *B. thailandensis* facilitated the uptake of Mn^2+^ by secreting a Mn^2+^-binding effector TseM during oxidative stress. The Mn^2+^ load on TseM could be delivered to MnoT, a Mn^2+^-specific TonB-dependent outer membrane transporter, and then transported into cells to fulfill the increased cellular demand for Mn^2+^ under oxidative stress (Si et al., 2017b). While in *P. aeruginosa*, a H3-T6SS secreted effector TseF (PA2374) is involved in iron uptake by interacting with outer membrane vesicles (OMVs) and the Pseudomonas Quinolone Signal (PQS) system (Lin et al., 2017). These studies highlight the newly characterized function of ion transport through T6SSs. Whether and how Yersinia T6SSs transport other metal ions besides Zn^2+^ needs to be investigated in the future.

### Other potential functions

More functions were identified in other bacterial species in addition to *Yersinia*. T6SS is also implicated in biofilm formation in several bacterial pathogens. For example, Hcp participates in biofilm formation in *P. aeruginosa* PAO1 (Southey-Pillig et al., 2005), and H1-T6SS is associated with biofilm-specific antibiotic resistance in *P. aeruginosa* PA14 (Zhang et al., 2011a). In *Acidovorax citrulli*, four mutants of T6SS components were reduced in biofilm formation (Tian et al., 2015). Recently, T6SS was shown to be associated with cell autophagy. VgrG2, a translocon of T6SS2 in *Vibrio parahaemolyticus*, induces autophagy in macrophages (Yu et al., 2015). A Type VI secretion PGAP1-like effector TplE, belonging to Tle4 phospholipase family in *P. aeruginosa* (Russell et al., 2013), induced autophagy through disruption of endoplasmic reticulum (ER) homeostasis (Jiang et al., 2016). In addition, the T6SS in *P. mirabilis* plays a role in self-recognition during swarming via delivery of toxic effectors (Alteri et al., 2013). Further research and analysis need to be performed to verify these potential T6SS functions in *Yersinia* species.

## Effectors secreted by T6SS in *yersinia*

With a bacteriophage tail-like structure, T6SS apparatus can deliver various effectors into bacterial competitors for interbacterial competition (Cianfanelli et al., 2016), into eukaryotic hosts for pathogenesis (Durand et al., 2014) or into extracellular milieu for nutritional demands (Wang et al., 2015; Lin et al., 2017; Si et al., 2017a). The antibacterial T6SS effector usually has a chaperone immunity protein that provides protection to the organism itself. These effectors could be divided into three categories as targeting the membrane, cell wall, or nucleic acid (Russell et al., 2014). The eukaryotic host-targeted T6SS effectors are diverse in biological and biochemical functions. For example, VgrG-1 in *V. cholerae* can cause actin crosslinking for intestinal inflammation (Ma and Mekalanos, 2010), VgrG2b in *P. aeruginosa* interacts with microtubules for successful invasion of epithelial cells (Sana et al., 2015), and VgrG-5 in *B. thailandensis* could induce the host cell membrane fusions by interacting directly with a neighboring host cell receptor or by engaging in homotypic interactions within or between cells (Schwarz et al., 2014). A recent publication summarizes the T6SS effectors identified in recent years (Lien and Lai, 2017).

Many T6SS secretion effectors have been identified in bacterial organisms. However, apart from the hallmark of T6SS effectors Hcp or VgrG, only two effectors were functionally characterized in *Yersinia* species according to published papers. They are YezP (YPK_3549) in *Y. pseudotuberculosis*, which is a novel Zn^2+^-binding protein substrate (Wang et al., 2015), and YPO0502 in *Y. pestis* CO92, which is a low-molecular-weight component with siderophore activity and regarded as the autoagglutination factor (Podladchikova et al., 2011). In addition, three T6SS associated proteins, y3673/Hcp, y3674 and y3675, were detected in the outer-membrane proteome in *Y. pestis* KIM6+ using 2DGE gel electrophoresis (Pieper et al., 2009). However, the y3674/y3675 genes possess VipB/VipA motif according to the KEGG database, which suggests these two proteins may comprise the contractile sheath of T6SS. With high-throughput signature-tagged mutagenesis (STM) approach, three T6SS components or effector-encoding genes (*vasK/ypo3603, ypo0498*, and *ypo1484*) were identified in *Y. pestis* CO92 (Ponnusamy et al., 2015). VasK was confirmed to be important during *Y. pestis* infection in mouse models of plague (Andersson et al., 2017). With a conserved “MxiM” motif, YPO0498 may be a lipoprotein, while YPO1484 is likely to be an RNase toxin with a sharp conical extension complex of “PAAR” (Proline-Alanine-Alanine-aRginine) (Shneider et al., 2013). However, more experiments need to be performed to explain the connection between these proteins and T6SS apparatus. Recently, Andersson et al generated three PAAR motif repeat-containing T6SS effector encoding gene deletion mutants (Δ*ypo0873*, Δ*ypo1484*, and Δ*ypo3615*). Only Δ*ypo1484* exhibited a limited level of attenuation, 20% survivability, in comparison to WT CO92 in a mouse model of pneumonic plague. This attenuation was similar to the level of attenuation, 14%, reported in the T6SS Cluster C (*ypo1458-1484*) deletion mutant, which contains the *ypo1484* gene (Andersson et al., 2017). As multiple copies of T6SS have been confirmed in *Yersinia* species, it seems that more potential undiscovered effectors remain to be identified. Here, we presented the hypothetical effectors with “PAAR” motif in *Yersinia* T6SS clusters (as shown in Table 2).

**Table 2:** Putative PAAR motif-containing effectors in *Yersinia* T6SS clusters.

	**T6SS Cluster**	**Predicted effectors with PAAR motif**	**Other Pfam**	**Function**	**HHpred**
*Y. pestis*	T6SS-A	YPO0511a	DUF4150, ScdA_N	Against oxidative/nitrosative stress	Tail-associated lysozyme
	T6SS-B	YPO0866		Bacterial membrane proteins	Tail-associated lysozyme
		YPO0873	Pyocin_S	Bacteriocin	S-type Pyocin family protein endonuclease activity
	T6SS-C	YPO1484	Ntox44	RNase toxin	Tail-associated lysozyme
	T6SS-G	YPO3615	RHS_repeat, RHS DUF4595	Bacterial membrane proteins	Tail-associated lysozyme
*Y. pseudotuberculosis*	T6SS1	YPK_0403	RHS_repeat, RHS DUF4595, Ntox47	ABC toxin complex	TccC3
	T6SS2	YPK_2589	Ntox44	RNase toxin	Tail-associated lysozyme PMID: 22731697
	T6SS3	YPK_0952	Pyocin_S	Bacteriocin	Tail-associated lysozyme, S-type pyocin
		YPK_0959		Bacterial membrane proteins	Endonuclease activity PMID: 12409205 Tail-associated lysozym
		YPK_1487		Bacterial membrane proteins	Tail-associated lysozyme
	T6SS4	YPK_3554	DUF4150, ScdA_N	Against oxidative/nitrosative stress	Tail-associated lysozyme
*Y. enterocolitica*	T6SS	YE2683a		Bacterial membrane proteins	Tail-associated lysozyme

## T6SS regulation in *yersinia*

T6SS gene clusters are found both in pathogenic and non-pathogenic bacteria, which are distributed in various environments including marine, soil, rhizosphere, higher plants and mammalian hosts, implying their diverse functions (Boyer et al., 2009). As the assembly, contraction, and disassembly cycle of the T6SS is likely to be energetically costly to the bacterial cell, the T6SS organelle is tightly regulated when organisms alternate in various environmental conditions (Silverman et al., 2012). It has been reported that T6SS in bacterial species responds to various environmental factors including salinity (Salomon et al., 2013), iron-concentration (Chakraborty et al., 2011), temperature (Pieper et al., 2009), QS-dependent cell density (Zheng et al., 2010; Kitaoka et al., 2011), host immune system, and other stressors (Brooks et al., 2013).

Temperature is a common environmental factor for most pathogens, and changes widely when organisms alternate in various environmental conditions. The expression of T6SS cluster A (*YPO0498-YPO0516* in *Y. pestis* CO92 or *y3658-y3677* in *Y. pestis* KIM) is induced by lowering the temperature from 37 to 26°C (Pieper et al., 2009; Robinson et al., 2009). In *Y. pseudotuberculosis*, all the four T6SS clusters are differentially thermoregulated. In addition to the induction of T6SS4 at 26°C, the T6SS1 expression is obviously induced at 37°C, while T6SS2, T6SS3, and T6SS4 was completely repressed at 37°C, which suggested that the T6SS1 played a more important role in bacterial virulence during mammalian host infection than other 3 T6SS loci. In addition, T6SS is precisely regulated by growth phase and acylated homoserine lactones (AHLs) dependent quorum sensing (QS) systems (Zhang et al., 2011b). Furthermore, T6SS4 in *Y. pseudotuberculosis* has been shown to respond to oxidative stress (Wang et al., 2015).

T6SS could be activated by different transcriptional regulators to combat multiple stresses and host immunity, and several transcriptional regulators have been identified in *Yersinia* species. In *Y. pseudotuberculosis*, the OmpR-regulated T6SS4 plays important roles under acidic and osmotic stress conditions (Gueguen et al., 2013; Zhang et al., 2013). It was proposed that T6SS4 was activated by RpoS and partly mediated the roles of RpoS in osmotic and acid resistance (Guan et al., 2015). Furthermore, T6SS4 could be regulated by OxyR or ZntR to cope with oxidative stress by removing ROS produced by host immunity or harmful environments (Wang et al., 2015, 2017). We have summarized the identified regulators of T6SS in *Yersinia* in Table 3.

**Table 3:** The identified transcriptional regulators for T6SSs in *Yersinia*.

**Regulator**	**Mode**	**Environment cues**	**T6SS copies of Organisms**	**References**
RovA	Positively/Indirectly	Host immunity	*Y. pestis* T6SS cluster A	Cathelyn et al., 2006
YpsI/YtbI (QS)	Positively/not sure	Growth phase	*Y. pseudotuberculosis* T6SS4	Zhang et al., 2011b
OmpR	Positively/directly	Acidic or osmotic stresses	*Y. pseudotuberculosis* T6SS4	Gueguen et al., 2013; Zhang et al., 2013
OxyR	Positively/directly	Oxidative stress	*Y. pseudotuberculosis* T6SS4	Wang et al., 2015
ZntR	Positively/directly	Oxidative stress	*Y. pseudotuberculosis* T6SS4	Wang et al., 2017
RpoS	Positively/directly	Osmotic, acid stresses	*Y. pseudotuberculosis* T6SS4	Guan et al., 2015
RovM	Positively/directly	Nutrition limited	*Y. pseudotuberculosis* T6SS4	Song et al., 2015

In addition to the research on *Yersinia*, other transcriptional regulators of T6SSs have been identified in more bacterial species. In *P. aeruginosa*, a T6SS is controlled by the global virulence regulator proteins RetS and LadS (Mougous et al., 2006). In *Salmonella enterica*, T6SS genes were controlled by the SsrA/SsrB two-component regulatory system (Parsons and Heffron, 2005). Furthermore, T6SS is regulated by AggR in enteroaggregative *Escherichia coli* (Dudley et al., 2006), by VirA/G in *Burkholderia mallei* (Schell et al., 2007), and similarly by quorum sensing mechanism in the plant pathogen *Pectobacterium atrosepticum* (Liu et al., 2008). By contrast, few post-transcriptional regulators have been identified so far. Lon, a posttranslational regulator, is known to affect a variety of physiological traits in many bacteria. It suggested that T6SS was regulated by LonA in *V. cholerae*, as well as biofilm formation, swimming motility, intracellular levels of cyclic diguanylate (Rogers et al., 2016). In *P. aeruginosa*, a screen to identify T6SS regulatory elements found that the posttranscriptional regulator RsmA imposes a concerted repression on all three T6SS clusters. RsmA and a transcriptional regulator AmrZ orchestrate the assembly of all three T6SSs in *P. aeruginosa* (Allsopp et al., 2017).

## Conclusions

**Figure 3:**
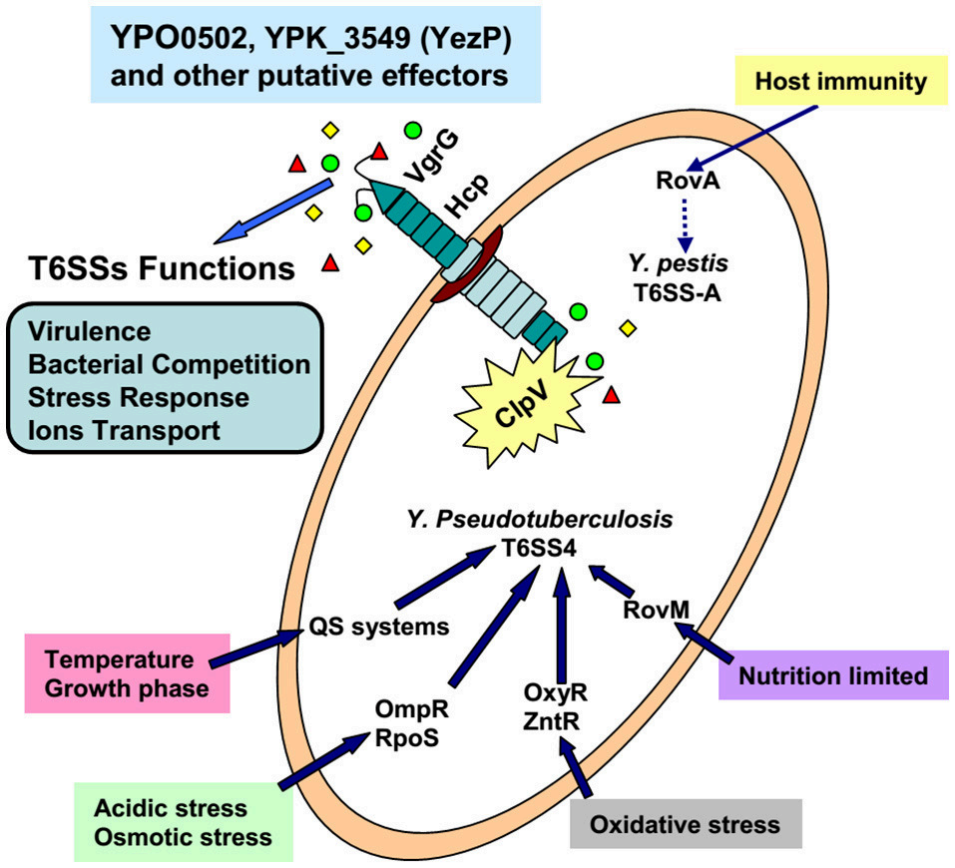
The confirmed environmental factors, transcriptional regulators, and functions of T6SSs in *Yersinia*.

Over the past decade, pathogenic bacteria *P. aeruginosa* and *V. cholerae* have been widely used to study T6SS, and significant advancements have been made on the understanding of their structure, activation signals, and regulatory pathways (Chen et al., 2015; Joshi et al., 2017). As important pathogenic bacteria, three *Yersinia* species also attracted increasing attention because of their multiple T6SS copies. As a summary, we drew a schematic diagram based on the current achievement of research on T6SSs in *Yersinia* (Figure 3). However, many questions need to be addressed. We believe that the future work in *Yersinia* T6SS research will involve at least six points: 1, The T6SS activation conditions; 2, The T6SS regulators and the regulation network; 3, Identification of T6SS secreted effectors and their new functions; 4, Based on the knowledge of T6SS attack and protection ability, the ways to prevent the T6SS^+^ pathogens; 5, The different functions and connection of different T6SS clusters; 6, The exploitation of new methods or techniques for further T6SS studies. The progress of *Yersinia* T6SS research will provide new knowledge and challenges, as well as further our understanding of the T6SS complexities in other important human pathogens. This research will also influence clinical medicine, microbial community ecology, and physiology.

## Author contributions

XY, JP, and YW collected and assessed the references; YW and XS contributed in the proposal and guideline of the review; XY, JP, and XS wrote the paper.

### Conflict of interest statement

The authors declare that the research was conducted in the absence of any commercial or financial relationships that could be construed as a potential conflict of interest.
